# p38 MAPK Regulates Expression of Immune Response Genes and Contributes to Longevity in C. elegans

**DOI:** 10.1371/journal.pgen.0020183

**Published:** 2006-11-10

**Authors:** Emily R Troemel, Stephanie W Chu, Valerie Reinke, Siu Sylvia Lee, Frederick M Ausubel, Dennis H Kim

**Affiliations:** 1 Department of Genetics, Harvard Medical School, Boston, Massachusetts, United States of America; 2 Department of Molecular Biology, Massachusetts General Hospital, Boston, Massachusetts, United States of America; 3 Department of Biology, Massachusetts Institute of Technology, Cambridge, Massachusetts, United States of America; 4 Department of Genetics, Yale University School of Medicine, New Haven, Connecticut, United States of America; 5 Department of Molecular Biology and Genetics, Cornell University, Ithaca, New York, United States of America; Stanford University School of Medicine, United States of America

## Abstract

The PMK-1 p38 mitogen-activated protein kinase pathway and the DAF-2–DAF-16 insulin signaling pathway control Caenorhabditis elegans intestinal innate immunity. *pmk-1* loss-of-function mutants have enhanced sensitivity to pathogens, while *daf-2* loss-of-function mutants have enhanced resistance to pathogens that requires upregulation of the DAF-16 transcription factor. We used genetic analysis to show that the pathogen resistance of *daf-2* mutants also requires PMK-1. However, genome-wide microarray analysis indicated that there was essentially no overlap between genes positively regulated by PMK-1 and DAF-16, suggesting that they form parallel pathways to promote immunity. We found that PMK-1 controls expression of candidate secreted antimicrobials, including C-type lectins, ShK toxins, and CUB-like genes. Microarray analysis demonstrated that 25% of PMK-1 positively regulated genes are induced by Pseudomonas aeruginosa infection. Using quantitative PCR, we showed that PMK-1 regulates both basal and infection-induced expression of pathogen response genes, while DAF-16 does not. Finally, we used genetic analysis to show that PMK-1 contributes to the enhanced longevity of *daf-2* mutants. We propose that the PMK-1 pathway is a specific, indispensable immunity pathway that mediates expression of secreted immune response genes, while the DAF-2–DAF-16 pathway appears to regulate immunity as part of a more general stress response. The contribution of the PMK-1 pathway to the enhanced lifespan of *daf-2* mutants suggests that innate immunity is an important determinant of longevity.

## Synopsis

The innate immune system provides the first line of defense against pathogen infection and relies upon pathways conserved across mammals, insects, and nematodes. Here, the authors have analyzed the transcriptional response of the nematode Caenorhabditis elegans to infection by the human pathogen Pseudomonas aeruginosa. They investigated this transcriptional response in the context of two conserved pathways involved in pathogen defense: the PMK-1 p38 mitogen-activated protein kinase (p38 MAPK) pathway and the DAF-2–DAF-16 insulin-signaling pathway. Specifically, the authors found that the p38 MAPK pathway plays a critical role in the infection-induced expression of secreted immune response genes. These genes include C-type lectins, lysozymes, and antimicrobial peptides that fight off infection in many species. In contrast, they found that the DAF-16 pathway is not required for immune response gene expression and may regulate immunity as part of a general stress response that functions in parallel to p38 MAPK. In addition, the authors observed that p38 MAPK contributes to the enhanced longevity of *daf-2* mutants, implicating p38 MAPK signaling in the regulation of longevity, possibly through its role in immunity.

## Introduction

The innate immune system constitutes the first line of defense in response to pathogen attack [1]. This “hard-wired” defense involves detection of pathogen-associated molecular patterns (PAMPs) that triggers upregulation of antimicrobial genes, as well as activation of more specific responses via the adaptive immune system. The innate immune system has ancient origins [2,3]; mammalian innate immunity uses molecular pathways conserved with flies and nematodes, even though these invertebrate animals lack classic adaptive immune systems.

The nematode Caenorhabditis elegans has been used as a model host for infection studies with the human opportunistic pathogen Pseudomonas aeruginosa to identify evolutionarily conserved mechanisms of innate immunity [2,4]. A forward genetic screen for immunocompromised mutants demonstrated a requirement for a conserved p38 mitogen-activated protein kinase (MAPK) pathway in C. elegans immunity [5]. This screen yielded mutants defective in NSY-1, a MAPK kinase kinase (MAPKKK) and in SEK-1, a MAPK kinase (MAPKK). These proteins signal via the PMK-1 p38 MAPK, as *pmk-1* mutants are also sensitive to pathogen killing, and the PMK-1 protein lacks activating phosphorylation in *nsy-1* and *sek-1* mutants. In mice, genetic analysis has recently demonstrated a similar role for ASK1, the mammalian NSY-1 homolog, in immunity [6]. ASK1 is required to activate p38 MAPK and downstream immune effectors in response to a bacterial PAMP, lipopolysaccharide. Thus, the NSY-1/SEK-1/PMK-1 cassette is an evolutionarily conserved module used in defense against pathogenic attack. However, the downstream effectors of C. elegans p38 MAPK signaling that promote pathogen resistance have not been identified.

In addition to the p38 MAPK pathway, another conserved pathway has been shown to regulate C. elegans innate immunity. The DAF-2 insulin/insulin-like growth factor receptor is a tyrosine kinase that was originally identified for its role in regulating entry into an alternative larval stage called dauer, and has subsequently been shown to have dramatic effects on lifespan and stress resistance [7–9]. Activation of the DAF-2 receptor ultimately results in phosphorylation of the DAF-16 FOXO/forkhead transcription factor [10,11], which then becomes sequestered in the cytoplasm, rendering it unable to regulate transcription. *daf-2* mutants are not only stress-resistant and long-lived, but also resistant to killing by the Gram-negative bacterial pathogen P. aeruginosa and other pathogens [12]. This resistance requires DAF-16, since the double-mutant *daf-2;daf-16* is not resistant to killing. Extensive microarray analysis has identified candidate downstream effectors of DAF-16 [13,14], including the lysozyme *lys-7,* which is a presumptive antimicrobial gene. *lys-7* had previously been identified in an analysis of the inducible defense response in C. elegans [15]. This study used RNA blot analysis to demonstrate that three lysozymes were upregulated by exposure to the Gram-negative bacterial pathogen *Serratia marcescens. daf-2* mutants may therefore be resistant to pathogens because DAF-16 is hyperactivated, causing increased expression of antimicrobial genes [13]. However, *daf-16* mutants exhibit similar sensitivity to pathogenic bacteria as wild-type animals [12], suggesting that the DAF-2–DAF-16 pathway is not important in wild-type worms for resistance to bacterial pathogens, at least as measured in the laboratory. Nothing is known about the interrelationship between the DAF-16 pathway and the PMK-1 p38 MAPK pathway that ultimately results in an immune response.

 In addition to the lysozymes described above, a limited number of other candidate C. elegans immune effectors have been identified. Seven S. marcescens–induced genes were identified using filter microarray analysis of a subset of the genome; three of these genes had homology to C-type lectins, which have recently been shown to have antibacterial activity and are induced by bacterial exposure in mice [16], and one gene had a CUB-like domain (formerly known as DUF141—domain of unknown function 141). A separate analysis of genes induced by the pathogenic fungus Drechmeria coniospora using filter microarrays identified several neuropeptide-like genes, including one with demonstrated antifungal activity [17]. Several other C. elegans genes have been proposed to function in pathogen defense based on their homology with known antimicrobials, such as Ascaris suum antibacterial factor [18] and amoebapore-like enzymes [19], including one with antibacterial activity that is upregulated by DAF-16. Programmed cell death in the germline has been shown to be induced by Salmonella enterica infection in a PMK-1–dependent manner, but the significance of this for immunity effector mechanisms is not clear [20,21]. Thus, expression, homology, and physiological studies have provided an initial sketch of the C. elegans repertoire of defense effectors.

More recently, comprehensive full-genome microarray approaches have begun to be applied to the C. elegans defense response. A full-genome analysis of the response to the pathogen Microbacterium nematophilum revealed a set of induced genes, including C-type lectins, lysozymes, and CUB-like genes [22]. In addition, a full-genome analysis of the transcriptional response to the bacterial pore-forming toxin Cry5B has also been reported [23]. However, an analysis of the transcriptional response to P. aeruginosa has not been described, and little is known about which pathways mediate the transcriptional response to pathogen attack.

The DAF-2–DAF-16 pathway has a role in both immunity and longevity. Functional analysis of presumptive antibacterial genes upregulated by DAF-16 indicated that some of these genes contribute to the increased lifespan of *daf-2* mutants [13]. These results are intriguing, as bacterial proliferation has been proposed to be a cause of death for *C. elegans.* Older animals have extensive intestinal proliferation of *E. coli,* their normal laboratory food source, and nematodes live longer on killed bacteria than on live bacteria [24,25]. Although these results suggest an important role for the innate immune response in regulating longevity, the role of immunity in determining lifespan is just beginning to be explored.

Here we describe genetic, genomic, and functional analyses of the PMK-1 and DAF-2–DAF-16 pathways. We integrate these results with a full-genome analysis of the response to pathogen infection to define how these pathways act in concert to regulate immunity and longevity. Our studies indicate that loss-of-function mutants in the PMK-1 pathway suppress the enhanced pathogen resistance to P. aeruginosa of *daf-2* mutants, suggesting that *pmk-1* is either downstream or in parallel to *daf-2.* Full-genome expression analysis suggests that the PMK-1 pathway acts in parallel to DAF-16, the key transcription factor downstream of DAF-2. PMK-1 controls expression of a suite of genes encoding secreted products that may act as antimicrobial genes and/or in cell–cell communication to promote immunity. We identified many of these same genes in a full-genome analysis of the C. elegans transcriptional response to *P. aeruginosa.* Using quantitative reverse transcription PCR (qRT-PCR) we found that PMK-1 mediates both basal and inducible expression of many infection response genes, while DAF-16 does not. In addition, we found that an apparent third immune pathway activated by P. aeruginosa infection functions independently of PMK-1 and DAF-16. Finally, consistent with immunity being an important determinant of longevity, we show that PMK-1 contributes to increased lifespan in *daf-2*, which identifies the PMK-1 p38 MAPK cassette as a regulator of lifespan.

## Results

### The PMK-1 p38 MAPK Cassette Is Required for the Enhanced Resistance to Pathogens of *daf-2* Mutants

**Figure 1:**
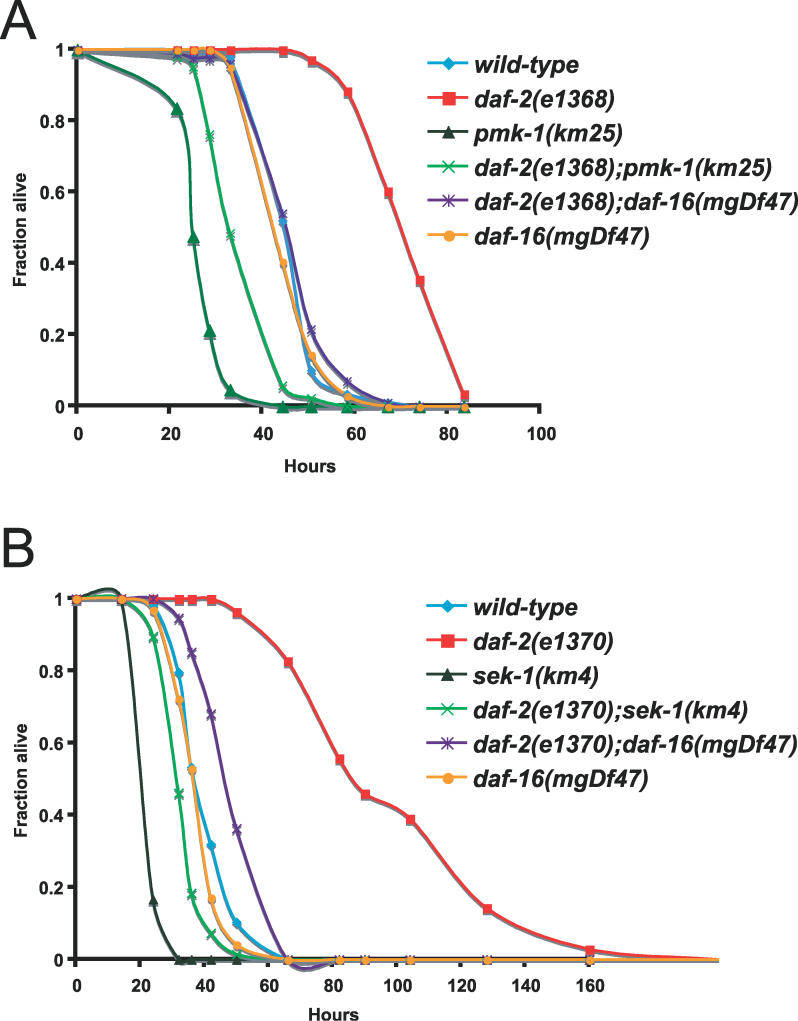
The PMK-1 p38 MAPK Pathway Is Required for *daf-2* Enhanced Pathogen Resistance (A) *pmk-1(km25)* suppresses *daf-2(e1368)* enhanced pathogen resistance. (B) *sek-1(km4)* suppresses *daf-2(e1370)* enhanced pathogen resistance. Slow killing assays were performed with P. aeruginosa strain PA14 under standard conditions, and each graph shows the average of three plates for each strain, with each plate containing 20–30 worms. Results are representative of 3 independent assays. See Table S1 for more assays and statistical analysis.

To examine the interaction between the PMK-1 p38 MAPK pathway and the DAF-2–DAF-16 insulin-signaling pathway, we constructed double mutants and analyzed their resistance to the pathogenic P. aeruginosa strain, PA14. As shown in Figure 1A, *daf-2(e1368)* partial loss-of-function mutants are more resistant to killing by P. aeruginosa than the wild-type N2 strain, while *pmk-1(km25)* deletion mutants are more sensitive to killing. *daf-2(e1368);pmk-1(km25)* double mutants are much more sensitive than *daf-2(e1368)* single mutants, indicating that wild-type PMK-1 is required for *daf-2(e1368)* enhanced pathogen resistance. These results are consistent with the PMK-1 p38 MAPK pathway acting downstream or in parallel to DAF-2. We confirmed these observations using a double mutant between *daf-2(e1370)* and a deletion in the MAPKK *sek-1*, which is upstream of *pmk-1* and is also required for pathogen resistance. Again, we find that the p38 MAPK pathway is required for *daf-2* pathogen resistance, as *daf-2(e1370);sek-1(km4)* double mutants are much more sensitive to killing by P. aeruginosa than *daf-2(e1370)* mutants (Figure 1B). Figures 1A and 1B also show that a *daf-16(mgDf47)* deletion mutant suppresses the resistance of *daf-2(e1368)* and *daf-2(e1370)* mutants, although *daf-16(mgDf47)* single mutants are not substantially more sensitive to pathogens than wild-type animals (see also Table S1), similar to the results with *daf-16(mgDf47)* mutants on the Gram-positive bacterial pathogens Enterococcus faecalis and Staphylococcus aureus [12]. A list of P. aeruginosa killing assay results and statistical analyses can be found in Table S1.

### The PMK-1 p38 MAPK Pathway Controls Expression of Secreted Genes

**Figure 2:**
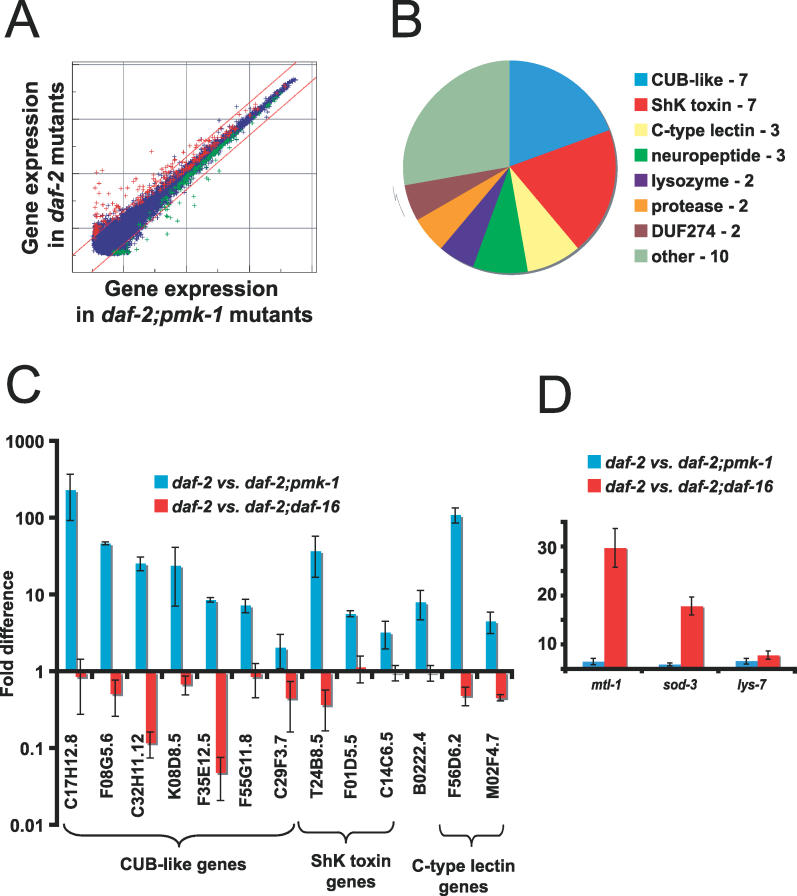
PMK-1 and DAF-16 Upregulate Distinct Genes (A) Intensity plot of genes upregulated by PMK-1 in *daf-2(e1368)* mutant. Plot represents signal intensity of all 22,500 sequences on Affymetrix C. elegans GeneChip. The *x*-axis shows expression level in *daf-2(e1368);pmk-1(km25)* animals and the *y*-axis shows expression level in *daf-2(e1368)* animals. Sequences colored red are considered upregulated by wild-type PMK-1 and sequences colored green are considered downregulated by wild-type PMK-1 (using Resolver; *p* <0.01). Diagonal lines delineate a 2-fold change. See Table S2 for a complete list of genes differentially expressed between *daf-2* and *daf-2;pmk-1*. (B) Pie chart of gene classes upregulated by PMK-1. The top 36 genes induced are represented. (C and D) qRT-PCR comparing expression in *daf-2(e1368)* versus *daf-2(e1368);pmk-1(km25),* and *daf-2(e1368)* versus *daf-2(e1368);daf-16(mgDf47).* Results are the average of two biological replicates, each replicate measured in duplicate and normalized to a control gene. Error bars are SEM. (C) shows genes upregulated by PMK-1 and (D) shows genes upregulated by DAF-16.

The pathogen resistance and longevity of *daf-2* mutants requires the DAF-16 transcription factor, as *daf-16* mutations suppress both the pathogen resistance and longevity of *daf-2* mutants. How does the PMK-1 pathway interact with the DAF-2–DAF-16 pathway? One possibility is that PMK-1 acts upstream of DAF-16, and regulates its transcriptional activity. Since *pmk-1* mutants are immunodeficient, while *daf-16* mutants are not, PMK-1 cannot rely solely on DAF-16, but DAF-16 might comprise one pathway downstream of PMK-1. If DAF-16 were one of the transcription factors that acts downstream of PMK-1, then one would expect to see similar genes regulated by PMK-1 and DAF-16. To address this possibility, we performed transcriptional profiling of PMK-1 to identify its downstream effectors and compare these with effectors downstream of DAF-16. In order to identify genes regulated by PMK-1, we used full-genome C. elegans oligonucleotide microarrays (GeneChips) from Affymetrix to compare *daf-2(e1368)* and *daf-2(e1368);pmk-1(km25)* animals feeding on E. coli OP50 harvested at the late larval/young adult stage. We identified 86 genes as upregulated more than 2-fold in *daf-2* compared to *daf-2;pmk-1.* These genes are thus upregulated by wild-type *pmk-1* (PMK-1). Figure 2A shows a graph of the expression intensity of all probe sets on the microarray in *daf-2(e1368);pmk-1(km25)* animals versus *daf-2(e1368)* animals. Genes upregulated by PMK-1 include C-type lectins, lysozymes, and neuropeptide-like genes (Figure 2B). The two most highly represented classes of PMK-1–upregulated genes were genes with a CUB-like domain and genes with homology to ShK toxins. There was a preponderance of secreted genes among PMK-1–regulated genes; based on Wormbase annotation, 31 of the top 36 genes upregulated by PMK-1 correspond to genes whose protein products are predicted to be secreted (Table 1). The fold regulation and *p*-values for these genes are listed in Table 1. We also found 44 genes downregulated greater than 2-fold. These genes included lectins, seven transmembrane domain receptors, and transporters. For a complete list of genes differentially regulated between *daf-2* and *daf-2;pmk-1,* see Table S2.

**Table 1:**
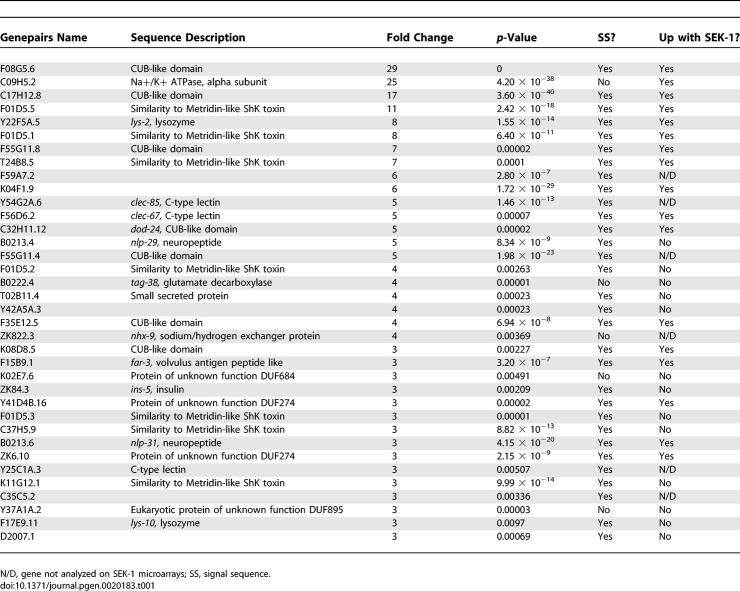
Genes Upregulated 3-Fold or More by Wild-Type PMK-1 in *daf-2* Mutants

We also carried out genome-wide microarray analysis to compare genes differentially expressed in *glp-4(bn2)* and *glp-4(bn2);sek-1(ag1)* mutants. We have previously shown that SEK-1 is required for PMK-1 activation, and that the *sek-1(ag1)* mutation confers enhanced pathogen susceptibility in the *glp-4(bn2)* background as well [5]. By utilizing sterile animals, these experiments excluded potential contributions from reproduction and examined differential gene expression regulated by the PMK-1 pathway in a genetic background distinct from *daf-2.* In this microarray analysis, we identified 101 genes as upregulated > 2-fold by SEK-1 and 6 genes downregulated > 2-fold by SEK-1 (Table S3). As is summarized in Table 1, we observed considerable overlap between the *glp-4(bn2)* versus *glp-4(bn2);sek-1(ag1)* microarray analysis and the *daf-2(e1368)* versus *daf-2(e1368);pmk-1(km25)* microarray analysis; notably, of the top ten PMK-1 upregulated genes, nine of them were also upregulated by SEK-1 (the tenth was not printed on the microarrays used for SEK-1 analysis).

### PMK-1 and DAF-16 Upregulate Distinct Genes

To address whether PMK-1 might upregulate gene expression via the DAF-16 transcription factor, we compared PMK-1–upregulated genes with published DAF-16–upregulated genes. We found very little overlap between our PMK-1–upregulated genes and DAF-16–upregulated genes identified by Murphy et al. (referred to by Murphy et al. as “Class I genes” [13]). C. elegans Affymetrix GeneChips had 240 Class I genes; however, only three Class I genes were upregulated by PMK-1 in our comparison. We also compared our data to data generated by McElwee et al [14]. Again, we found very little overlap between PMK-1–upregulated and DAF-16–upregulated genes—we only found two of the 453 genes upregulated more than 2-fold with DAF-16 in this dataset were also upregulated with PMK-1 (these two genes are different from the three genes found in the comparison with Murphy et al. data). However, we found significant overlap between PMK-1–upregulated genes and DAF-16–downregulated genes (see Materials and Methods for calculation of overlap expected by chance). Thirteen of the PMK-1–upregulated genes were considered to be downregulated by DAF-16 in Murphy et al. microarrays, whereas only one gene would be expected by chance. Similarly, 28 PMK-1–upregulated genes were also considered to be downregulated by DAF-16 in McElwee et al. microarrays, whereas only about four genes would be expected by chance.

In order to confirm this comparative analysis that was based on microarray experiments done in different labs and with different microarray platforms, we also generated our own microarray data using Affymetrix GeneChips. We compared *daf-2(e1368)* and *daf-2(e1368);daf-16(mgDf47)* strains to identify DAF-16–regulated genes. Again, we found very little overlap between PMK-1–upregulated genes and DAF-16–upregulated genes. Only five genes were upregulated by both PMK-1 and DAF-16 (84 genes upregulated by DAF-16), and these genes did not have large fold changes in either comparison. However, we found significant overlap between genes predicted to be downregulated by DAF-16 and upregulated by PMK-1, including CUB-like genes. Fourteen genes were found to be both upregulated by PMK-1 and downregulated by DAF-16, and these genes had large fold changes (only two genes would be expected by chance).

As an independent confirmation of our microarray experiments, we performed qRT-PCR expression analysis of 13 genes identified as being upregulated by PMK-1 in microarray experiments. We compared expression of these genes in *daf-2(e1368)* versus *daf-2(e1368);daf-16(mgDf47)* and *daf-2(e1368)* versus *daf-2(e1368);pmk-1(km25)* (Figure 2C). In agreement with microarray data, we found that all the PMK-1–upregulated genes were either downregulated or not regulated by wild-type DAF-16 (Figure 2C). This expression analysis confirms our microarray results and indicates that DAF-16 and PMK-1 upregulate mutually exclusive sets of genes to promote immunity. We also confirmed that these 13 genes were induced by PMK-1 in a wild-type background (Figure S1). Therefore, many of the genes identified by microarrays as upregulated by PMK-1 in a *daf-2* background are also likely to be upregulated in a wild-type background. Because PMK-1–upregulated genes in general were not more highly expressed in *daf-2* mutants compared to wild-type (data not shown), we support a model where PMK-1 acts in parallel to both DAF-2 and DAF-16, its downstream transcription factor.

As a further examination of whether PMK-1 and DAF-16 might upregulate genes in common, we performed qRT-PCR on genes upregulated by DAF-16, as identified in our microarrays, to determine whether PMK-1 might also upregulate them. We tested three genes: *mtl-1,* because this gene had the greatest fold change; *sod-3,* because it is a commonly used marker for DAF-16 activity [26]; and *lys-7,* since this gene has been implicated in the immune response. (All three of these genes were also considered to be upregulated by DAF-16 by Murphy et al. [13]) While we confirmed that DAF-16 does upregulate these genes, we did not find a requirement for PMK-1 (Figure 2D). In addition, we found that RNA interference (RNAi) to *pmk-1* or *sek-1* did not affect nuclear localization of DAF-16::GFP in a *daf-2(e1370)* mutant (unpublished data). These data suggest that PMK-1 does not regulate transcription through DAF-16.

### Gene Expression and Pathogen Resistance of *pmk-1;daf-16* Double Mutants

The analyses described above indicated that PMK-1 and DAF-16 upregulate different genes, suggesting that PMK-1 and DAF-16 act in parallel. To further examine whether this is true, we generated animals that have mutations in both *pmk-1* and *daf-16* and measured the effect on gene expression in mutant animals feeding on *E. coli.* If these genes act in parallel, then gene expression in double mutants should be the sum of each of the single mutants. We generated *daf-16(mgDf47);pmk-1(km25)* double-mutant animals as well as *daf-2(e1368);daf-16(mgDf47);pmk-1(km25)* triple-mutant animals and tested the expression of genes that are both upregulated by PMK-1 and downregulated by DAF-16. As would be expected if PMK-1 and DAF-16 acted in parallel, expression of these genes was at an intermediate level in these double and triple mutants, apparently due to the sum of opposing effects from PMK-1 and DAF-16 (Figure S2). For example, expression of C32H11.12 had low expression in *pmk-1* mutants (because it is turned up by wild-type PMK-1), high expression in *daf-16* mutants (because it is turned down by wild-type DAF-16), and intermediate expression in *daf-16;pmk-1* mutants (because PMK-1 and DAF-16 have opposing effects).

If PMK-1 and DAF-16 act in parallel to promote immunity, *daf-16;pmk-1* double mutants should be more sensitive to pathogens than *pmk-1* single mutants. To test this, we measured survival of *daf-16;pmk-1* double mutants and found that they were more sensitive to PA14 than *pmk-1* single mutants (see Table S1). In addition, we tested *daf-2;daf-16;pmk-1* triple mutants and found that they were more sensitive to PA14 than *daf-2;pmk-1* double mutants. These results are consistent with PMK-1 and DAF-16 acting in parallel to promote immunity.

### Rapid Transcriptional Changes in Response to Pathogenic P. aeruginosa

**Figure 3:**
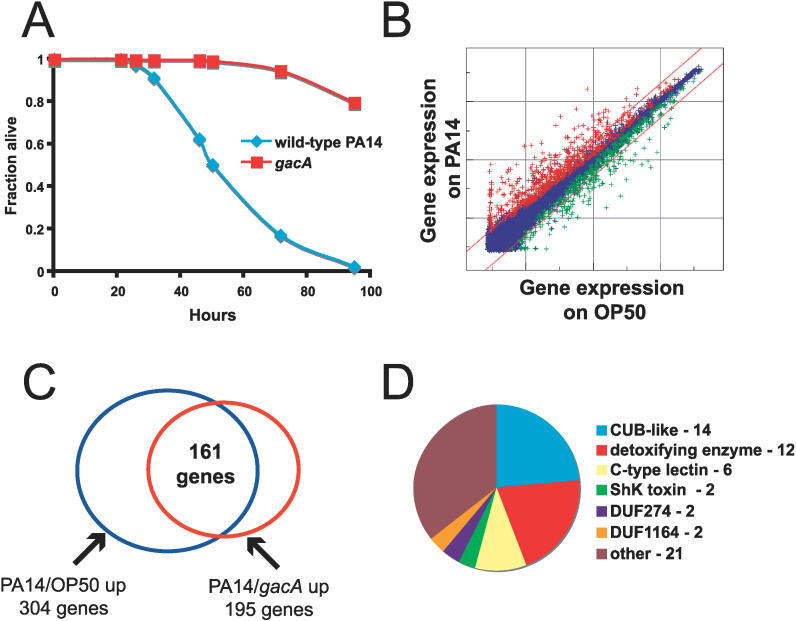
P. aeruginosa Induces Expression of Genes in Similar Gene Classes as PMK-1–Induced Genes (A) PA14 slow killing assay comparing survival of *fer-15;fem-1* young adult animals on *gacA* and wild-type PA14. Animals were raised in the same way as for harvest for microarrays. (B) Intensity plot of genes regulated by PA14 versus OP50 at 4 h. Sequences colored red are considered upregulated by PA14 and sequences colored green are considered downregulated by wild-type PA14 (using Resolver; *p* <0.01). Diagonal lines delineate a 2-fold change. See Table S4 for a complete list of genes differentially expressed between PA14 and OP50. (C) Venn diagram of overlap between genes induced in PA14 versus OP50 at 4 h comparison and PA14 versus *gacA* at 4 h comparison. (D) Pie chart of gene classes induced by PA14 versus OP50. The genes (59) that are upregulated greater than 5-fold are represented.

The microarray and qRT-PCR studies described above were not performed on worms exposed to pathogen, but rather on worms grown on E. coli strain OP50, which is commonly used as a food source for *C. elegans.* Since both the PMK-1 pathway and the DAF-2–DAF-16 pathway are important for immunity, we wondered whether pathogens might induce the same set of genes as those regulated by PMK-1 or by DAF-16. Therefore, we used Affymetrix full-genome GeneChips to profile the transcriptional response to P. aeruginosa strain PA14. To avoid problems with pathogen-induced embryo retention that might affect gene expression, we used the temperature-sensitive sterile strain *fer-15(b26);fem-1(hc17),* which has been validated for other expression profiling studies, such as the analysis of genes regulated by DAF-16 [13] and by starvation [27]. We compared the gene expression of *fer-15(b26);fem-1(hc17)* animals exposed to 3 different bacteria, either E. coli strain OP50 (their normal food source), the pathogenic P. aeruginosa strain PA14, or an isogenic PA14 mutant *gacA* [28], which is less pathogenic to the C. elegans strain *fer-15(b26);fem-1(hc17)* (Figure 3A). We then harvested animals 4 and 8 h after transfer to the bacteria, extracted RNA, and examined gene expression using Affymetrix microarrays.

We found a rapid and robust transcriptional response to *P. aeruginosa.* At 4 h after exposure, we found 304 genes upregulated more than 2-fold in response to wild-type P. aeruginosa compared to *E. coli,* and 114 genes downregulated more than 2-fold (intensity plot in Figure 3B). At 8 h after exposure, we found slightly fewer genes upregulated (261 genes) and somewhat more genes downregulated (216 genes).

To address concerns that some of the regulated genes may be responding to the difference in bacterial species, rather than the pathogenicity of bacteria, we also compared expression on wild-type PA14 to expression on the less pathogenic *gacA* PA14. At 4 h after exposure, we found 195 genes upregulated more than 2-fold in response to wild-type P. aeruginosa compared to *gacA* mutants. Most (161) of these 195 genes were also considered to be upregulated in our first comparison (304 genes upregulated on P. aeruginosa compared to E. coli) (Figure 3C). This significant overlap indicates that the majority of genes upregulated on P. aeruginosa compared to E. coli reflect pathogenicity-induced changes. However, because *gacA* mutants are still pathogenic compared to *E. coli,* we focused our analysis on the genes identified as differentially regulated between wild-type PA14 and E. coli to be more inclusive. See Tables S4 and S5 for a complete list of genes regulated by P. aeruginosa infection.

### Overlap between Genes Induced by P. aeruginosa and Genes Induced by other Pathogens and by Toxins

In order to investigate the specificity of the transcriptional response to P. aeruginosa infection, we compared our dataset to published microarray datasets analyzing responses to other pathogens. Seven genes were described as upregulated by S. marcescens in a filter-based array study [15]. These genes included a CUB-like gene and C-type lectins, but they were different from the CUB-like genes and C-type lectins induced by *P. aeruginosa.* Only one S. marcescens–induced gene, *cnc-2,* was also upregulated by P. aeruginosa at 4 h. This gene was also among the six genes induced by D. coniospora infection [17], suggesting that it is a general response gene. D. coniospora studies also identified neuropeptide genes, but the exact genes appear to be different from our P. aeruginosa studies.

We also compared our results to a recent study of M. nematophilum infection [22], which is the first full-genome analysis of the C. elegans response to pathogen infection. M. nematophilum infection occurs through a mechanism distinct from other bacterial pathogens, and a different MAPK pathway (the extracellular signal–regulated kinase pathway) is required for response to this infection [29]. Despite these differences, there was substantial overlap between M. nematophilum–induced genes and the P. aeruginosa–induced genes—of the 68 genes induced by M. nematophilum infection, 23 were also induced by P. aeruginosa infection.

In order to determine what fraction of the P. aeruginosa–induced genes may be responding to intestinal damage, we compared these genes to those upregulated by exposure to the bacterial pore-forming toxin, Cry5B, and the heavy-metal toxin, cadmium [23]. We found substantial overlap between these datasets—97 of the 370 genes upregulated by Cry5B were also upregulated by P. aeruginosa versus OP50 at 4 h. Of the 388 genes induced by cadmium exposure, 98 genes were also induced by P. aeruginosa infection. Thus, some of the transcriptional response to P. aeruginosa infection is likely to be a response to intestinal damage.

### Overlap between Genes Regulated by PMK-1 and by P. aeruginosa

The gene classes induced by P. aeruginosa were very similar to those induced by PMK-1 (Figure 3D). Genes upregulated included the secreted CUB-like genes, ShK toxins, and C-type lectins. In addition, a significant number of genes that may function as detoxifying enzymes were identified, such as glutathione-S-transferases, acetyl-transferases, and UDP-glucuronosyl transferases [30]. These enzymes can add water-soluble moeities onto toxins to aid in their excretion, or otherwise inactivate them.

**Figure 4:**
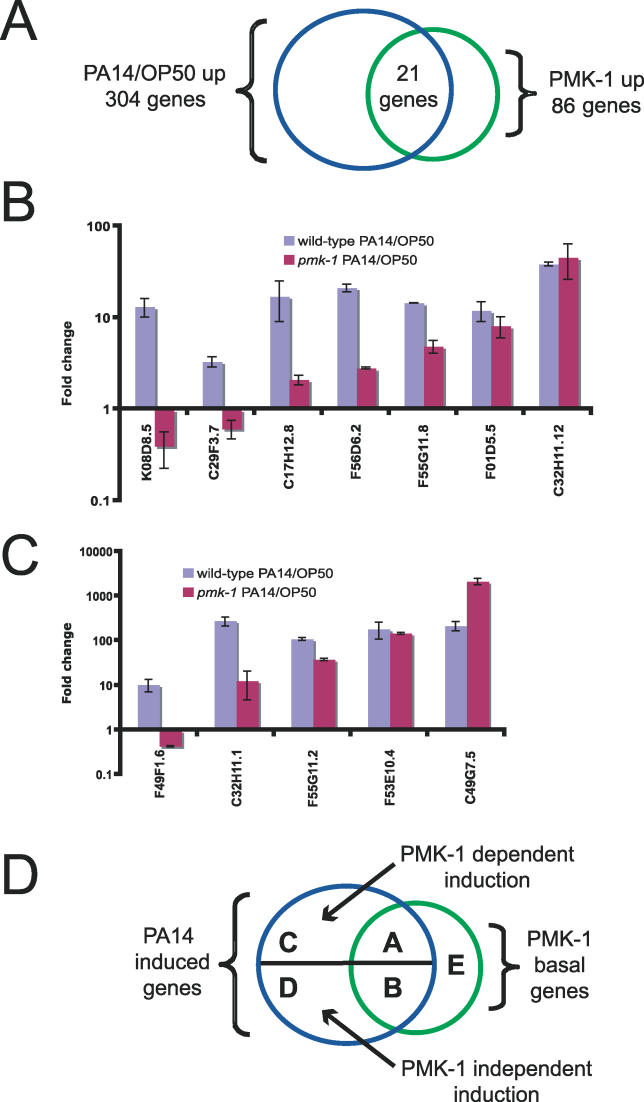
PMK-1 Regulates Basal and Inducible Expression of P. aeruginosa–Induced Genes (A) Venn diagram of overlap between genes regulated by PMK-1 and *P. aeruginosa.* (B) (C) qRT-PCR analysis of PA14-induced gene expression in wild-type animals and in *pmk-1* mutants. Results are the average of two biological replicates, each replicate measured in duplicate and normalized to a control gene. Error bars are SEM. (D) Diagram of different gene classes regulated by PMK-1 and/or *P. aeruginosa.* PMK-1 is required for basal and inducible regulations of class A genes. PMK-1 is required for basal, but not inducible expression of class B genes. PMK-1 is required for inducible but not basal expression of class C genes. PMK-1 is required for neither basal nor inducible expression of class D genes. PMK-1 regulates basal expression of class E genes, but these genes are not induced by *P. aeruginosa.*

Of the 304 genes upregulated more than 2-fold by *P. aeruginosa,* 21 genes were also upregulated more than 2-fold by PMK-1 in the microarrays comparing *daf-2* and *daf-2;pmk-1* (Figure 4A). Therefore, about 25% of PMK-1–upregulated genes are induced by PA14 infection. This overlap is significant because an overlap of only about 1 gene would be expected by chance alone. In order to investigate the functional significance of these genes (hereafter referred to as overlap genes) as well as other PMK-1–upregulated genes, we used RNAi to knock down their expression and then tested resistance to *P. aeruginosa.* We tested 18 overlap genes as well as 20 other PMK-1–induced genes (see Table S6 for a list of genes tested by RNAi). However, none of the genes inactivated individually by RNAi reproducibly resulted in an enhanced susceptibility to pathogens (unpublished data). One possible explanation for the lack of phenotype observed in these experiments is that these genes exhibit extensive redundancy, and removal of only one gene alone does not cause immunodeficiency. Another possibility is that there was incomplete knockdown of these genes by RNAi.

Because the genes regulated by PMK-1 on E. coli were identified in microarrays using a *daf-2(e1368)* mutant background, and the genes induced by PA14 were identified in microarrays using a *fer-15(b26);fem-1(hc17)* background, we wanted to confirm that these genes are regulated similarly in a wild-type background. We had already confirmed that ten of the overlap genes were indeed upregulated by PMK-1 in a wild-type background on E. coli (Figure S1). We next tested seven of these overlap genes for induction by P. aeruginosa compared to E. coli in wild-type animals 4 h after exposure. All seven genes that we examined using qRT-PCR were induced by P. aeruginosa in wild-type animals (Figure 4B), in agreement with our microarray data. Therefore, many of the genes identified from our microarray studies using different strain backgrounds are also likely to be regulated similarly in a wild-type background.

### PMK-1 Regulates both Basal and Inducible Expression of Pathogen Response Genes

The qRT-PCR analysis described above confirmed that basal expression of the overlap genes requires PMK-1 in animals grown on *E. coli,* and that these genes are induced by infection with *P. aeruginosa.* But does this induction require PMK-1? To ask whether PMK-1 is required for P. aeruginosa induction of overlap genes, we examined P. aeruginosa–induced gene expression in *pmk-1* mutants. We found that most of the overlap genes were not fully induced by P. aeruginosa in *pmk-1(km25)* mutants, including some that were not induced at all, indicating that PMK-1 is required for their induction (Figure 4B). However, some induced genes did not require PMK-1, even though they require PMK-1 for their basal expression (e.g., *C32H11.12;* baseline regulation of *C32H11.12* by PMK-1 is shown in Figure S1). Therefore, it appeared that the basal expression of some genes on *E. coli,* such as *C32H11.12,* is regulated by PMK-1, whereas the P. aeruginosa–induced expression of these genes requires a separate pathway.

The experiments above describe analyses of genes whose basal expression is regulated by PMK-1, and which are induced by pathogen attack. But some P. aeruginosa–induced genes do not require PMK-1 for their basal expression on *E. coli,* based on microarray data. Do these genes require PMK-1 for their induction by P. aeruginosa? We tested induction of the top five genes upregulated by P. aeruginosa versus E. coli at 4 h (see Table S4) and found that PMK-1 was required for induction of one of them and partially required for two of them (Figure 4C). The remaining two genes *(C49G7.5* and *F53E10.4)* must therefore be induced via a PMK-1–independent pathway, similar to *C32H11.12* (Figure 4B).

Together, the microarray and qRT-PCR studies of genes regulated by PMK-1 and *P. aeruginosa* identify five classes of candidate immunity genes (Figure 4D). Class A genes are regulated basally by PMK-1 on *E. coli,* and require PMK-1 for their induction by pathogenic P. aeruginosa (e.g., *K08D8.5*). Class B genes are regulated basally by PMK-1 and are induced by *P. aeruginosa,* but that induction does not require PMK-1 (e.g., *C32H11.12*). Class C genes are not regulated basally by PMK-1 based on microarray results, but do require PMK-1 for induction by P. aeruginosa (e.g., *F49F1.6*). Class D genes are not regulated basally by PMK-1 based on microarray results and do not require PMK-1 for induction by P. aeruginosa (e.g., *C49G7.5*). Class E genes are regulated basally by PMK-1 but are not induced *by P. aeruginosa.*

### DAF-16 and Genes Induced by P. aeruginosa Infection

The results described above strongly implicate the PMK-1 pathway in regulation of the transcriptional response to P. aeruginosa infection. Next, we examined the connection between DAF-16 and the response to *P. aeruginosa.* We compared genes upregulated by DAF-16 in our *daf-2* versus *daf-2;daf-16* microarrays, which were performed on *E. coli,* with genes induced by *P. aeruginosa.* We only found two genes that were upregulated more than 2-fold by wild-type DAF-16 on E. coli and induced more than 2-fold by P. aeruginosa infection. However, many of the genes induced more than 2-fold by P. aeruginosa infection were downregulated by DAF-16 (70 of a total of 445 genes downregulated by DAF-16). To determine whether DAF-16 might play a role in induction of genes by *P. aeruginosa,* we treated *daf-16(mgDf47)* mutants with P. aeruginosa and used qRT-PCR to assess gene induction. All ten genes tested were still induced in *daf-16(mgDf47)* mutants, indicating that DAF-16 does not mediate their induction (Figure S3). Furthermore, we did not see a DAF-16::GFP translational fusion enter the nucleus upon exposure to *P. aeruginosa,* although it did enter the nucleus upon heat stress (unpublished data and [31]). Therefore, we did not find any evidence that DAF-16 plays an active role in the transcriptional response to *P. aeruginosa.* Moreover, since DAF-16 is not responsible for the PMK-1–independent induction of Class B or D genes, we thus conclude that a third independent immune pathway must mediate this response.

### The p38 MAPK Cassette Is Partially Required for the Increased Lifespan of *daf-2* Mutants

**Figure 5:**
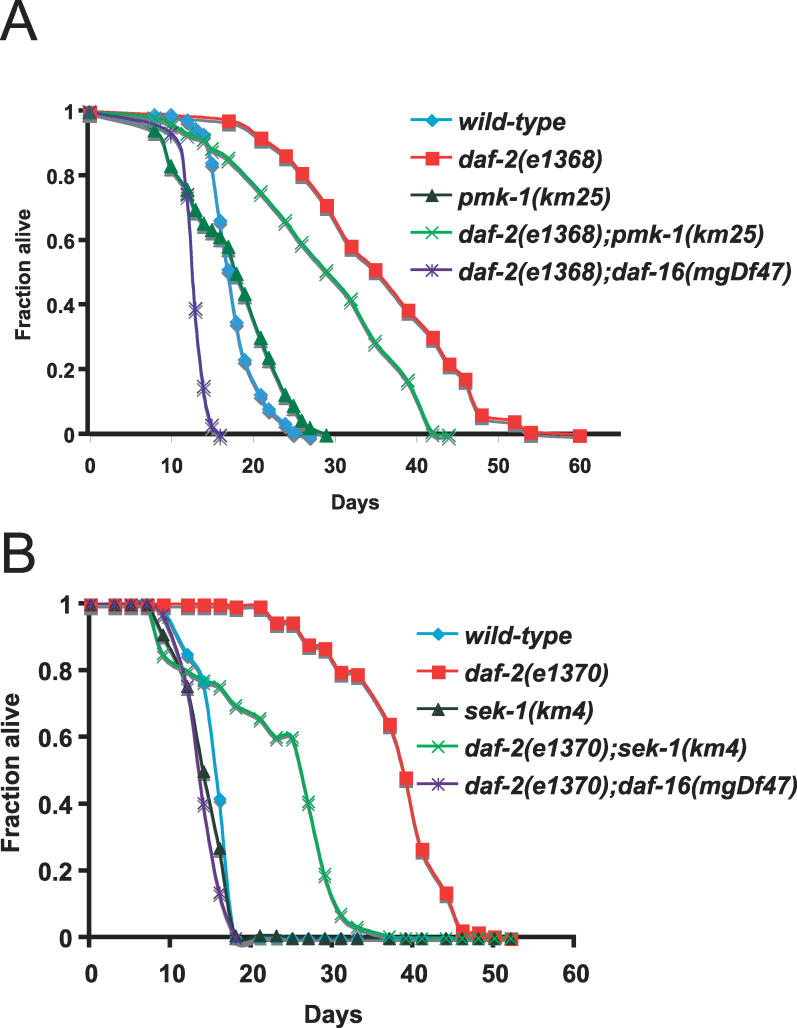
The PMK-1 p38 MAPK Pathway Is Partially Required for Increased Longevity of *daf-2* (A) *pmk-1(km25)* partially suppresses enhanced longevity of *daf-2(e1368)*. (B) *sek-1(km4)* partially suppresses enhanced longevity of *daf-2(e1370)*. See Table S7 for quantitative data.

Our genetic analysis of the PMK-1 p38 MAPK pathway demonstrated that it is required for the pathogen resistance of *daf-2* mutants. Because the *daf-2* pathway is a central regulator of longevity, we asked whether the p38 MAPK pathway is also required for the enhanced longevity of *daf-2* mutants. We tested whether inhibition of the PMK-1 pathway would affect the extended longevity of *daf-2* mutants on the relatively nonpathogenic E. coli strain OP50. This analysis showed that *pmk-1(km25)* partially suppressed the extended lifespan phenotype of *daf-2(e1368)* mutants (Figure 5A) as well as *daf-2(e1370)* mutants (Table S7). RNAi knock down of *pmk-1* similarly suppressed the extended lifespan phenotype of *daf-2* mutants (Table S7), suggesting that the lifespan suppression caused by loss of *pmk-1* is not allele specific. Furthermore, *daf-2(e1370);sek-1(km4)* mutants have shortened longevity compared to *daf-2(e1370)* mutants (Figure 5B), indicating that both the MAPKK and the MAPK in this pathway affect longevity. Importantly, the single *sek-1* or *pmk-1* null mutants exhibited a relatively normal lifespan on OP50 in the majority of experiments ([5] and Table S7). These mutants did show a slightly shortened lifespan in some experiments that were performed at 25 °C, but a slightly extended lifespan in some experiments performed at 20 °C. These results suggest that the *sek-1* or *pmk-1* mutation does not cause nonspecific illness. Because loss of the PMK-1 pathway suppressed the enhanced lifespan of *daf-2* mutants at both 20 °C and 25 °C in all the experiments tested (see Figure 5 and Table S7), we concluded that a functional PMK-1 pathway is important for the extended longevity of the *daf-2* mutants. While the null mutations in *pmk-1* and *sek-1* were able to suppress the effects of *daf-2* mutations on pathogen resistance and lifespan, they did not suppress the dauer constitutive phenotype of *daf-2* mutants (unpublished data), demonstrating specificity to their effects.

## Discussion

### PMK-1 and DAF-16 Play Distinct Roles in Controlling C. elegans Immunity

**Figure 6:**
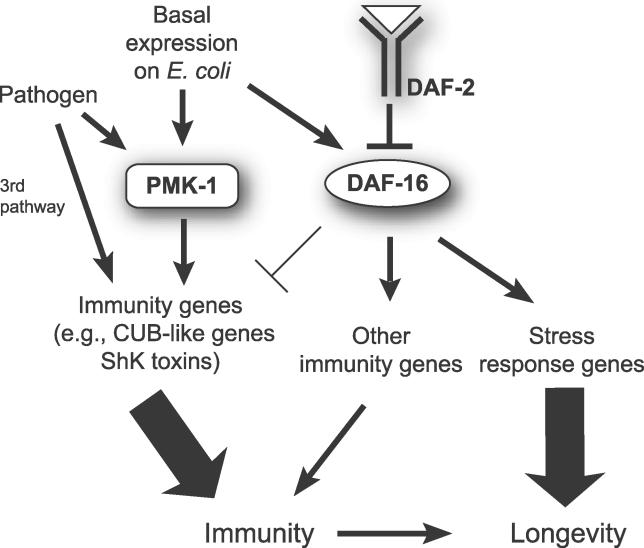
A Model for Regulation of Immunity/Longevity by the PMK-1 p38 MAPK Pathway and the DAF-2–DAF-16 Insulin-Signaling Pathway in C. elegans We propose that the PMK-1 p38 MAPK pathway plays an active role in response to bacterial pathogens. PMK-1 controls basal levels of pathogen response genes on E. coli and also mediates induction of these genes upon infection. In contrast, DAF-16 controls a separate group of immune genes and does not mediate the infection response, but does turn down expression of some pathogen-induced genes. Certain pathogen response genes are induced independently of PMK-1 or DAF-16, indicating the existence of a third pathway. PMK-1 is required for the enhanced pathogen resistance of *daf-2* mutants because it is the predominant pathway contributing to immunity. Finally, we suggest that PMK-1 contributes to enhanced longevity of *daf-2* mutants because intact immune function promotes longevity.

Our analysis of the PMK-1 pathway, the DAF-2–DAF-16 pathway, and the transcriptional response to pathogen infection provides a model for immunity and longevity in C. elegans (Figure 6). The PMK-1 and DAF-16 pathways appear to act in parallel to promote immunity; we found virtually no overlap between genes positively regulated by PMK-1 and genes positively regulated by DAF-16 when animals are feeding on *E. coli.* Our analysis of genes induced by pathogen infection showed that about 25% of the genes positively regulated by PMK-1 on E. coli were also induced by infection with the pathogen *P. aeruginosa.* In contrast, virtually none of the genes positively regulated by DAF-16 on E. coli was induced by *P. aeruginosa.* Layered on top of this “basal” expression of immune-related genes when C. elegans is feeding on E. coli is a role for PMK-1 in induction of pathogen response genes. Many of the genes induced by P. aeruginosa required PMK-1 for their induction, while none of the genes we examined required DAF-16 for their induction. We also found that a significant number of genes were induced in the absence of either PMK-1 or DAF-16, suggesting that a third immune pathway (or pathways) regulates their induction by *P. aeruginosa.* We investigated two candidates to test whether they might act in this third pathway (*dbl-1* of the TGF-β pathway and *kgb-1* of the JNK kinase pathway). Our initial experiments with selected genes suggested that *dbl-1* and *kgb-1* do not regulate pathogen-induced gene expression (unpublished data). Further experiments will be required to determine the nature of this third pathway.

Genetic analysis of the interaction between the PMK-1 pathway and the DAF-2–DAF-16 pathway demonstrated that PMK-1 is required for the enhanced resistance of *daf-2* mutants. Since PMK-1 appeared to act in parallel to DAF-2–DAF-16, we hypothesize that the PMK-1 pathway is so critical for immunity that even when DAF-16 is hyperactivated (like in *daf-2* mutants), loss of the PMK-1 pathway results in pathogen sensitivity. The relative contributions of PMK-1 and DAF-16 to immunity are illustrated in Figure 6.

The observation that DAF-16 and PMK-1 act in parallel is somewhat surprising, as DAF-16 and PMK-1 superficially appear to act in similar ways. Both are required for the enhanced immunity of *daf-2* mutants, and both contribute to the enhanced longevity of *daf-2* mutants. However, there are important differences between the functions of these genes. PMK-1 is critical for immunity in both a *daf-2* and a wild-type background. In contrast, while DAF-16 is critical for the enhanced immunity of *daf-2* mutants, it is not required for immunity in a wild-type background. Under well-fed conditions on E. coli in a wild-type background, DAF-16 is mostly inactive in the cytoplasm, due to repression from DAF-2 [31,32]. We also saw that DAF-16 was mostly cytoplasmic in animals on *P. aeruginosa.* Consistent with this, we saw no evidence for DAF-16 being involved in the inducible transcriptional response to *P. aeruginosa.* Therefore, we favor a model in which upregulation of DAF-16 in a *daf-2* mutant background activates a nonspecific response that promotes resistance to the pathogen *P. aeruginosa,* but in which DAF-16 plays no significant role in specific responses to this pathogen.

How might these parallel PMK-1 and DAF-16 pathways eventually converge on immune function? We have shown that PMK-1 functions in a specific immune response pathway, while DAF-2–DAF-16 appears to play a more general, nonspecific role. We found no evidence that DAF-16 responds to P. aeruginosa infection, despite its well-known role in acute stress responses [7]. Therefore, an intriguing hypothesis is that in nature, the contribution of DAF-16 to immunity is via an acute stress pathway, which is only revealed in the lab in a *daf-2* mutant. Work in mammals suggests that acute stress can boost the immune system [33,34]. Perhaps DAF-16 mimics the effect of acute stress to generally boost pathogen resistance in *C. elegans.* However, this boost is not sufficient to maintain immune responses in the absence of the indispensable PMK-1 pathway.

We found a number of genes upregulated by PMK-1 and pathogen infection that were downregulated by DAF-16, such as the CUB-like genes. If the CUB-like genes function to promote immunity, then DAF-16 would act to dampen this immune response. The mammalian DAF-16 homolog, FOXO3a, has been shown to inhibit certain immune responses: *foxo3a^−/−^* knockout mice have increased lymphocyte proliferation, inflammation, and cytokine overproduction [35]. However, in *C. elegans,* the observation that DAF-16 is required for *daf-2* mutant–enhanced pathogen resistance strongly supports the idea that DAF-16 promotes certain immune responses. An interesting parallel situation may be found in the role of mammalian FOXO3a in cell survival; some FOXO3a-regulated genes promote cell survival, while other genes promote apoptosis [36,37]. Perhaps FOXO transcription factors have opposing roles in immunity as well, acting to promote certain kinds of immunity, but to inhibit others. This subtle downregulation of certain immune response genes by DAF-16 may account for some of its lifespan-enhancing effects on *E. coli.* Immune response proteins may be energetically costly to make and not necessary when growing on a relatively nonpathogenic food source.

### The PMK-1 Pathway Regulates Expression of Secreted Candidate Antimicrobials in Response to P. aeruginosa Infection

Our expression analysis has identified a group of genes positively regulated by PMK-1. The proteins encoded by the majority of these genes are likely to be secreted. These proteins may function as antimicrobial peptides secreted into the intestinal lumen, or as signaling molecules used for cell–cell communication. Thus, the outputs of C. elegans p38 MAPK signaling have similarities with mammalian p38 MAPK signaling, which controls production of secreted antimicrobials and inflammatory cytokines in response to lipopolysaccharide [38]. One of the most common gene classes upregulated by PMK-1 is the class of genes with CUB-like domains. The CUB-like gene family comprises a group of 50–60 C. elegans genes, which are found in clusters in the genome and have been hypothesized to have a role in pathogen response [39]. The function of CUB-like domains is not known, but their sequence is related to CUB domains, which are found in many functionally diverse proteins. CUB domains are normally found extracellularly and have been found in many proteases, including complement components of the mammalian immune system [40]. Another prominent gene class upregulated by PMK-1 and P. aeruginosa includes C-type lectins. A recent study demonstrated that these genes are induced in the mouse intestine in response to bacterial exposure, and that they have antibacterial properties [16]. It will be interesting to determine whether p38 MAPK regulates expression of C-type lectins in the mammalian intestine as well.

The profiling of genes induced upon exposure to P. aeruginosa compared to E. coli after 4 h identified a rapid transcriptional response to this pathogen. Using qRT-PCR we found some genes were upregulated more than 100-fold at this early time point. Because most of these genes had lower expression on the less-pathogenic mutant *gacA* compared to wild-type *P. aeruginosa,* we believe that the bulk of the P. aeruginosa versus E. coli profile represents a response to pathogenicity, and not to some other difference between these bacterial strains, such as nutritional quality.

What are the PAMPs detected by C. elegans that trigger this transcriptional response? Classical PAMPs, such as bacterial cell wall components and flagellin, might not induce this particular response, as it differentiates between virulent and avirulent bacteria of the same strain. Therefore, virulence factors, or the effect of virulence factors, might act as “PAMPs” for *C. elegans.* However, it is also possible that pathogenic bacteria simply have more access to the C. elegans intestine, and the increased exposure of classical PAMPs from these bacteria is the relevant cue. A comparison of genes induced by P. aeruginosa with genes induced by the pore-forming toxin Cry5B or by cadmium revealed a substantial amount of overlap, suggesting intestinal damage triggers induction of many of these genes. However, approximately half of the genes induced by P. aeruginosa have not been reported in any of the other microarrays to which we compared our data (*S. marcescens, D. coniospora, M. nematophilum,* Cry5B, and cadmium). Further work will be required to determine the nature of PAMPs detected by C. elegans that trigger this immune response.

### The PMK-1 p38 MAPK Pathway Contributes to the Enhanced Longevity of *daf-2*

The DAF-16 FOXO transcription factor has been shown to regulate lifespan in C. elegans and *Drosophila.* Our genetic dissection of the PMK-1 p38 MAPK pathway demonstrated that it also regulates lifespan. Studies in C. elegans and *Drosophila* have demonstrated a role for a different MAPK pathway, the JNK pathway, in promoting longevity [41,42]. Perhaps the p38 pathway functions similarly to the JNK pathway? Both *Drosophila* and C. elegans studies indicate that the effects of JNK on lifespan are via the DAF-16 transcription factor [42,43]. However, our genomic analysis of genes regulated by PMK-1 and DAF-16 suggests that this is not the case for the p38 MAPK pathway.

How might PMK-1 contribute to the increased longevity of *daf-2* mutants? Some of the increased longevity of *daf-2* mutants mediated by DAF-16 appears to act via DAF-16 upregulated antimicrobials. Perhaps PMK-1 plays a similar role via distinct immune effectors to enhance lifespan (Figure 6). Immunity may play an important role in human longevity as well. Human aging does not occur in a sterile environment, but in the context of thousands of potentially pathogenic microbes. For example, the human gut is colonized by more than 500 species of bacteria [44], and an adult human has about ten times more microbial cells than human cells [45]. There is a strong association between immune function and individual human lifespan [46]. Furthermore, death due to infections such as pneumonia, influenza, nephritis, and septicemia are among the ten top causes of death for people over 65 years old [47]. Further defining the interplay between immunity and longevity in C. elegans may provide us with general insights into the mechanisms of human aging and age-related disease as well.

## Materials and Methods

### C. elegans strains.

All strains were maintained on nematode growth media (NGM) and fed with E. coli strain OP50, as described [48]. Mutations used in this study include: *daf-2(e1368), daf-2(e1370), pmk-1(km25), daf-16(mgDf47),* and *sek-1(km4). pmk-1(km25)* mutants were kindly provided by the Matsumoto lab [49]. Strain CF512: *fer-15(b26);fem-1(hc17)* was kindly provided by the *Caenorhabditis* Genetics Center (CGC; http://www.cbs.umn.edu/CGC). Strain TJ356: DAF-16::GFP was kindly provided by Sean Curran in the Ruvkun lab at Massachusetts General Hospital, and *daf-2(e1370);DAF-16::GFP* was kindly provided by Javier Irazoqui in the Ausubel lab at Massachusetts General Hospital.

### Pathogen assays.

Slow-killing P. aeruginosa assays were performed with the strain PA14 as described [28]. All assays were performed with L4 stage hermaphrodites unless noted. Briefly, PA14 was cultured in Luria broth, seeded on slow-kill plates, which contain modified NGM (0.35% instead of 0.25% peptone), and incubated first for 24 h at 37 °C and then for 24 h at 25 °C before adding worms. A total of 20–30 L4 stage worms were transferred to each pathogen plate and a total of at least three plates per strain were tested for each experiment. All killing assays were conducted at 25 °C. Worms were scored by gentle prodding with a platinum wire to test for live or dead animals.

### Lifespan assays.

Gravid adult worms were allowed to lay eggs for about 12 h onto NGM plates seeded with OP50. These eggs were allowed to grow to adults at 20 °C or 25 °C. About 20–30 adult animals were then transferred to each NGM plate seeded with OP50 and containing 0.05–0.1 mg/ml of 5-fluorodeoxyuridine (FUDR), which is added to prevent progeny from hatching. A total of three to four plates were tested for each strain in each experiment. Similar conditions were used for RNAi lifespan experiments, except that gravid adult worms were allowed to lay eggs onto plates seeded with RNAi bacteria. The progeny were allowed to grow to adults on RNAi bacteria and were transferred to FUDR-containing plates that were seeded with freshly induced RNAi bacteria. The animals were then kept at 20 °C or 25 °C, and scored every 2 d by gentle prodding with a platinum wire to test for live or dead animals. Lifespan is defined as the time elapsed from when worms were put on FUDR plates (adult lifespan = 0) to when they were scored as dead. Worms that died of protruding/bursting vulva, bagging, or crawling off the agar were censored. Statistical analyses were performed using the software SPSS (SPSS, http://www.spss.com). The survival experience of each mutant population was compared to that of the wild-type population using the log-rank test. A *p*-value <0.05 was considered significantly different from control.

### C. elegans growth and collection for *pmk-1*, *daf-16,* and P. aeruginosa microarray analysis using Affymetrix GeneChips.

*Microarrays to identify genes regulated by PMK-1 and by DAF-16. daf-2(e1368), daf-2(e1368);pmk-1(km25),* and *daf-2(e1368);daf-16(mgDf47)* animals were synchronized by hypochlorite treatment and first larval stage (L1) arrest. Arrested L1s were placed onto 10-cm NGM plates seeded with OP50 and grown at the *daf-2*–permissive temperature (15–16 °C) until L4, then shifted to the *daf-2*–restrictive temperature (25 °C) for 6 h, and then harvested. Three separate replicates of each strain were isolated. *daf-2* and *daf-2;pmk-1* animals grew at the same rate, but *daf-2;daf-16* animals were not perfectly synchronous with *daf-2.* In two preps *daf-2;daf-16* animals were slightly younger than *daf-2* animals (preps A and B), and in the third prep, *daf-2;daf-16* animals were slightly older than *daf-2* animals (prep C).

*Microarrays to identify pathogen regulated genes. fer-15(b26)ts;fem-1(hc17)* animals were synchronized by hypochlorite treatment and L1 arrest. Arrested L1s were placed onto NGM plates seeded with OP50 and grown at the restrictive temperature (25 °C) in order to obtain sterile adults. After ~52 h of growth at 25 °C (young adulthood), animals were transferred to slow-kill plates seeded either with OP50, wild-type PA14, or *gacA* mutant PA14. Animals were harvested at 4 and 8 h after transfer. Three independent replicates of each treatment were isolated.

*RNA isolation.* Total RNA was extracted using TRI Reagent (Molecular Research Center, http://www.mrcgene.com) according to the manufacturer's instructions, followed by purification on an RNeasy column (Qiagen, http://www1.qiagen.com).

### Microarray target preparation and hybridization for Affymetrix GeneChips.

RNA samples were prepared and hybridized on Affymetrix full-genome GeneChips for C. elegans at the Harvard Medical School Biopolymer Facility, according to instructions from Affymetrix (http://www.affymetrix.com). Briefly, 5 μg of total RNA was reverse transcribed using an oligo dT-T7 primer and Superscript II reverse transcriptase, followed by second-strand cDNA synthesis. The double-stranded cDNA was then purified with a column and was used as the template for in vitro transcription using T7 RNA polymerase and biotinylated nucleotides. The resulting cRNA was fragmented and hybridized onto C. elegans GeneChips.

### Microarray analysis for *pmk-1*, *daf-16* and P. aeruginosa studies (Affymetrix GeneChips).

Affymetrix .cel files were uploaded into the Resolver Gene Expression Data Analysis System, version 5.1 (Rosetta Inpharmatics, http://www.rii.com) at the Harvard Center for Genomic Research (http://www.cgr.harvard.edu) for analysis. For each condition, three replicate microarrays were normalized and combined using the Resolver intensity error model for single color chips [50,51]. Different conditions were then compared in Resolver to determine fold change for each probe set and a *p*-value, using a modified *t* test. Genes with a 2-fold or greater fold change and a *p*-value <0.01 were considered differentially expressed.

### C. elegans growth and collection for *sek-1* analysis using cDNA microarrays.

Parallel synchronized populations of *sek-1(ag1)* and *glp-4(bn2);sek-1(ag1)* animals were generated by hypochlorite treatment and L1 arrest, followed by propagation on 10-cm NGM plates seeded with OP50 and grown at 20 °C until the worms reached the L4 stage, at which point the worms were grown at 25 °C for an additional 24 h prior to harvesting and freezing of worms. No eggs were laid, and no progeny was observed. RNA isolation was carried out using a RNAzol reagent in combination with a homogenizer. Enrichment for mRNA was carried out using oligo-dT beads (Ambion, http://www.ambion.com), and at least 10 μg of polyA RNA was isolated. Worm propagation, harvesting, and RNA isolation was carried out independently for each of the three microarray experiments.

### Microarray analysis for *sek-1* studies (cDNA microarrays).

Fluorescence-labeled cDNA probes for DNA microarray hybridization were prepared from 5 μg of mRNA as described [52]. *glp-4* cDNA was labeled with Cy5 and compared to *glp-4;sek-1* cDNA labeled with Cy3. C. elegans whole-genome microarrays were used for hybridization as described [53]. Each slide was scanned using an Axon scanner (Molecular Devices, http://www.moleculardevices.com), and the expression levels for each gene in each channel were collected using GenePix 3.0 software. Cy5/Cy3 ratios were calculated and normalized by setting the overall median of ratios to one. To determine which genes changed in response to SEK-1 signaling, we calculated the mean log ratio of *glp-4/glp-4;sek-1,* and used a two-tailed, unpaired Student's *t* test, without correction for multiple testing, to identify genes with statistically significant changes. Genes with a 2-fold difference or greater and a *p*-value <0.05 were considered to be candidate SEK-1 targets.

### Comparisons of different microarray datasets.

In order to determine the overlap between microarray datasets expected by chance alone, we found the fraction of genes regulated in dataset 1, and then multiplied this by the number of genes regulated in dataset 2. For example, PMK-1 upregulated 0.43% of the genes in the genome [(86 genes upregulated) / (20,000 genes in the genome)], and there were 231 DAF-16 downregulated genes from Murphy et al. Therefore, the expected overlap by chance would be about 1 gene [(0.0043 × 231 genes) = 1.032 genes].

### Quantitative RT-PCR (qRT-PCR) analysis.

Animals were treated as described for samples used in microarray analysis. All strains compared were grown in parallel. (For comparisons of *daf-2* and *daf-2;daf-16* animals, which grow at different rates, four independent preps were compared. In some preps *daf-2;daf-16* were slightly younger than *daf-2* animals, and some preps *daf-2;daf-16* were slightly older than *daf-2* animals. We did not find a substantial difference in gene expression between the *daf-2;daf-16* preps of slightly different ages for the genes we analyzed). Total RNA was then extracted using TRI Reagent, and reverse transcribed using the Retroscript kit (Ambion). This cDNA was then subjected to qRT-PCR analysis using SYBR green detection on an iCycler machine (Bio-Rad, http://www.bio-rad.com). Primers for qRT-PCR were designed using Primer3 (Massachusetts Institute of Technology), checked for specificity against the C. elegans genome and tested for efficiency with a dilution series of template. All values are normalized against control genes that do not vary under conditions being tested (*snb-1* primers are the control for *pmk-1* and *daf-16* comparisons and *nhr-23* primers [27] are the control for pathogen treatment comparisons). Fold change is calculated using the Pfaffl method [54]. Primer sequences are available upon request.

### Feeding RNAi experiments to test PMK-1–regulated genes.

RNAi of selected genes regulated by PMK-1 was carried out using the *daf-2(e1368)* and *eri-1 (mg366)* mutants and bacterial feeding RNAi [55]. L4 animals were transferred to RNAi plates and their L4 progeny were transferred to plates seeded with PA14 as described previously. *daf-2(e1368)* mutants were propagated at 15 °C, and shifted to 25 °C 12 h prior to transfer as L4 stage larvae to PA14 plates. Plates were analyzed in triplicate, and repeated on different days and compared. RNAi clones were obtained from the Ahringer [56] and Vidal [57] laboratories, and sequences of the RNAi clones were confirmed by sequencing.

## Supporting Information

Figure S1 Gene Expression in N2 versus *pmk-1(km25)* and N2 versus *daf-16(mgDf47)*
qRT-PCR was used to determine relative expression of 13 different genes. Results are the average of two biological replicates, each replicate measured in duplicate and normalized to a control gene. Error bars are SEM. “O” denotes a gene that is an overlap gene (i.e., also is induced by P. aeruginosa on microarrays). “i” denotes a gene that was demonstrated to be induced by P. aeruginosa with qRT-PCR in Figure 4B.
(561 KB PDF)

Figure S2 Analysis of PMK-1–Upregulated Genes in Mutants Defective in *pmk-1* and *daf-16*
(A) Gene expression in N2 versus *pmk-1(km25)*, N2 versus *daf-16(mgDf47)*, and in N2 versus *daf-16(mgDf47);pmk-1(km25)*.
(B) Gene expression in *daf-2(e1368)* versus *daf-2(e1368);daf-16(mgDf47), daf-2(e1368)* versus *daf-2(e1368);pmk-1(km25)*, and in *daf-2(e1368)* versus *daf-2(e1368);daf-16(mgDf47);pmk-1(km25)*.
qRT-PCR was used to determine relative expression of five different genes. Results are the average of 2 biological replicates, each replicate measured in duplicate and normalized to a control gene. Error bars are SEM.
(569 KB PDF)

Figure S3 PA14-Induced Gene Expression in N2 and *daf-16(mgDf47)* Mutants qRT-PCR was used to determine pathogen-induction of ten different genes. Results are the average of two biological replicates, each replicate measured in duplicate and normalized to a control gene. Error bars are SEM.
(528 KB PDF)

Table S1 Effects of *pmk-1* and *sek-1* Mutations on P. aeruginosa PA14 Killing SPSS software was used to calculate mean survival and *p*-values using the log-rank test. All *sek-1* assays were performed with ≥12 h shift to the *daf-2* restrictive temperature prior to the start of the assay. *pmk-1* assays were performed with a 6- to 12-h temperature shift prior to the start of the assay, as noted for each assay.
(22 KB XLS)

Table S2 Genes Differentially Expressed between *daf-2* and *daf-2;pmk-1*
This table lists all Affymetrix probe sets called differentially expressed between *daf-2* and *daf-2;pmk-1* animals (>2-fold change, *p* <0.01).
(43 KB XLS)

Table S3 Genes Differentially Expressed between *glp-4* and *glp-4;sek-1*
This table lists all cDNAs called differentially expressed between *glp-4* and *glp-4;sek-1*animals (>2-fold change, *p* <0.05).
(32 KB XLS)

Table S4 Genes Differentially Expressed between Exposure to E. coli Strain OP50 and Wild-Type P. aeruginosa Strain PA14 This table lists all Affymetrix probe sets called differentially expressed between animals exposed to OP50 versus PA14 (>2-fold change, *p* <0.01). One worksheet contains the data for 4 h, the other for 8 h.
(194 KB XLS)

Table S5 Genes Differentially Expressed between Exposure to *gacA* Mutant and Wild-Type P. aeruginosa Strain PA14 This table lists all Affymetrix probe sets called differentially expressed between animals exposed to wild-type versus *gacA* mutant PA14 (>2-fold change, *p* <0.01). One worksheet contains the data for 4 h, the other for 8 h.
(143 KB XLS)

Table S6 PMK-1–Upregulated Genes that Were Treated with RNAi and Tested for Resistance to PA14 (21 KB XLS)

Table S7 Effects of *pmk-1* and *sek-1* Mutations on Lifespan SPSS software was used to calculate mean survival and *p*-values using the log-rank test.
(16 KB XLS)

### Accession Numbers

The Wormbase (http://www.wormbase.org) accession numbers for C. elegans genes and gene products discussed in this paper are *pmk-1* (B0218.3), *sek-1* (R03G5.2), *daf-2* (Y55D5A.5), *daf-16* (R13H8.1), and *cnc-2* (R09B5.3). The entire dataset from all microarray experiments can be downloaded from the National Center for Biotechnology Information Gene Expression Omnibus (GEO; http://www.ncbi.nlm.nih.gov/geo). The National Center for Biotechnology GEO accession numbers for the comparisons discussed in this paper are P. aeruginosa and E. coli comparisons (GSE5793) and *daf-2, daf-2;daf-16* and *daf-2;pmk-1* comparisons (GSE5801).

## Abbreviations

K: kinase
L: larval stage
MAPK: mitogen-activated protein kinase
NGM: nematode growth medium
PAMP: pathogen-associated molecular pattern
qRT-PCR: quantitative reverse transcription PCR
RNAi: RNA interference

## Note Added in Proof

Shapira et al. [58] have reported a microarray analysis of genes induced by P. aeruginosa infection and identified many of the same genes described in this study.
