# Supplementary material for: p38 MAPK Regulates Expression of Immune Response Genes and Contributes to Longevity in C. elegans
Source: PLoS Genet. 2006 Nov 10;2(11):e183. doi: 10.1371/journal.pgen.0020183 (PMC1635533; doi:10.1371/journal.pgen.0020183)
Supplement: Supplementary file 1 [file pgen.0020183.sg001.pdf]

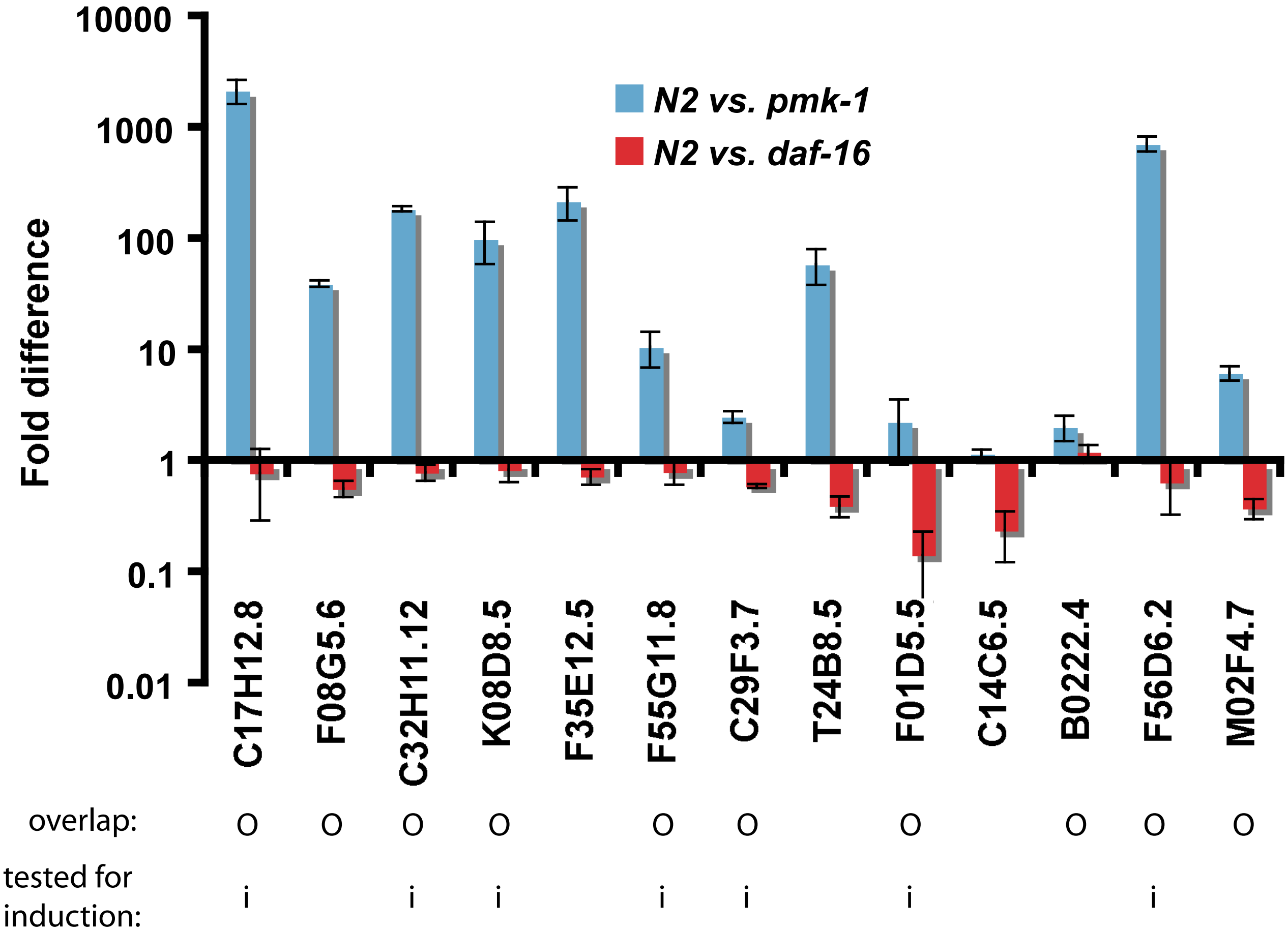

10000
1000
100
10
1
0.1
0.01
Fold difference
N2 vs. pmk-1
N2 vs. daf-16
C17H12.8
F08G5.6
C32H11.12
K08D8.5
F35E12.5
F55G11.8
C29F3.7
T24B8.5
F01D5.5
C14C6.5
B0222.4
F56D6.2
M02F4.7
overlap:
O O O O O O O O O O
tested for
induction:
i i i i i i i
