# Supplementary material for: p38 MAPK Regulates Expression of Immune Response Genes and Contributes to Longevity in C. elegans
Source: PLoS Genet. 2006 Nov 10;2(11):e183. doi: 10.1371/journal.pgen.0020183 (PMC1635533; doi:10.1371/journal.pgen.0020183)
Supplement: Supplementary file 2 [file pgen.0020183.sg002.pdf]

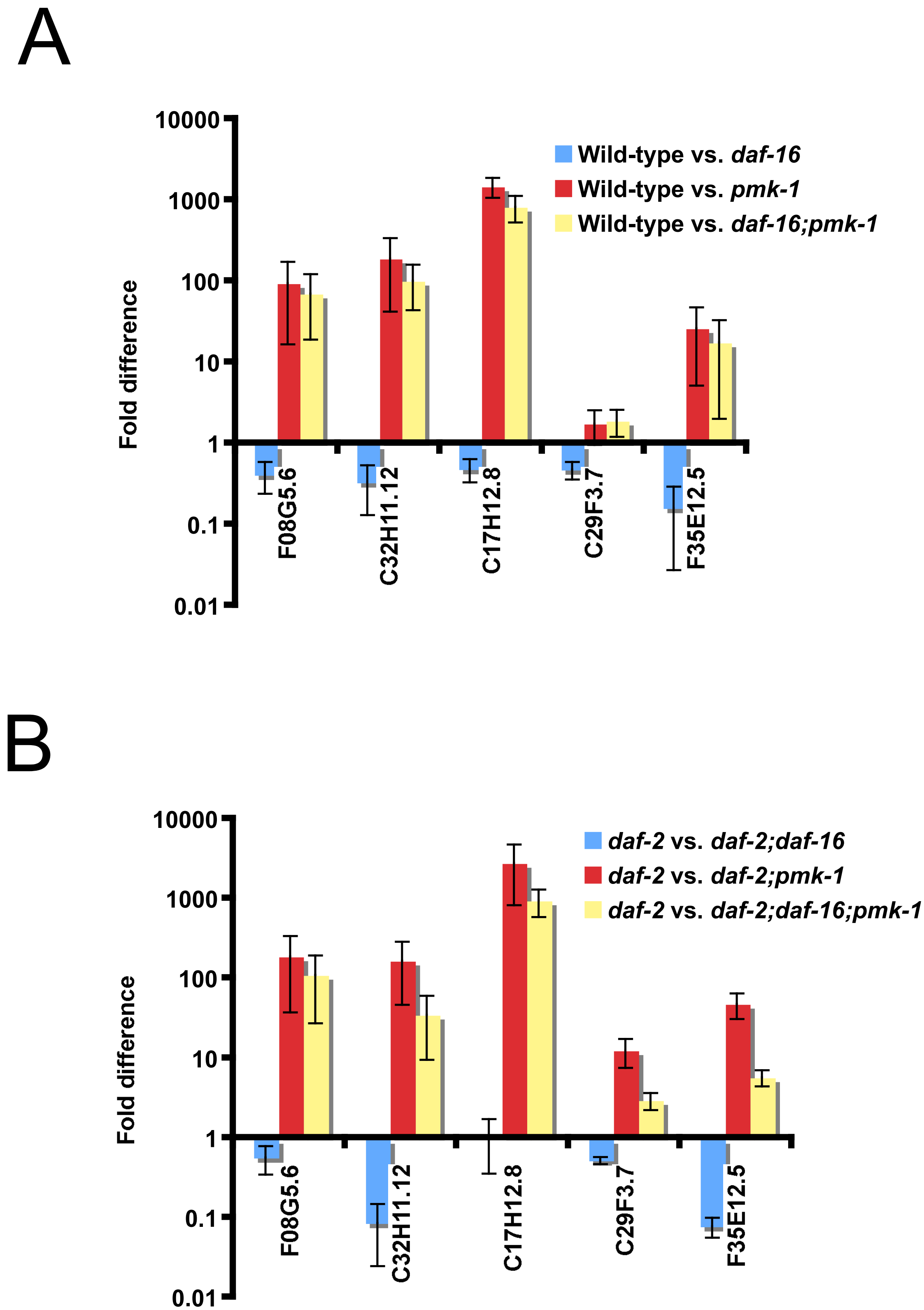
A
10000
1000
100
10
1
0.1
0.01
Fold difference
Wild-type vs. *daf-16*
Wild-type vs. *pmk-1*
Wild-type vs. *daf-16;pmk-1*
F08G5.6
C32H11.12
C17H12.8
C29F3.7
F35E12.5
B
10000
1000
100
10
1
0.1
0.01
Fold difference
daf-2 vs. *daf-2;daf-16*
daf-2 vs. *daf-2;pmk-1*
daf-2 vs. *daf-2;daf-16;pmk-1*
F08G5.6
C32H11.12
C17H12.8
C29F3.7
F35E12.5
