# Supplementary material for: p38 MAPK Regulates Expression of Immune Response Genes and Contributes to Longevity in C. elegans
Source: PLoS Genet. 2006 Nov 10;2(11):e183. doi: 10.1371/journal.pgen.0020183 (PMC1635533; doi:10.1371/journal.pgen.0020183)
Supplement: Supplementary file 3 [file pgen.0020183.sg003.pdf]

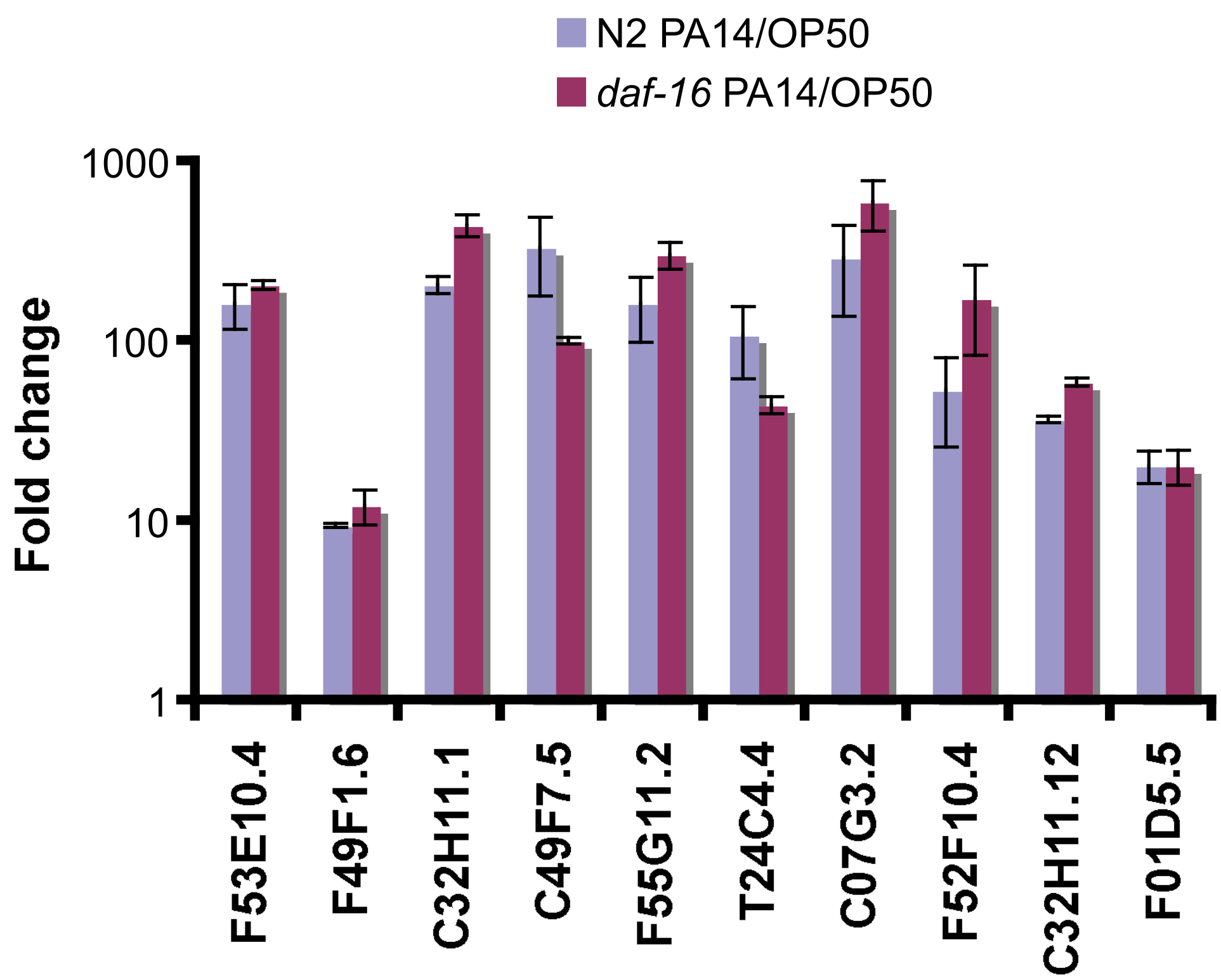
N2 PA14/OP50
daf-16 PA14/OP50
Fold change
1000
100
10
1
F53E10.4
F49F1.6
C32H11.1
C49F7.5
F55G11.2
T24C4.4
C07G3.2
F52F10.4
C32H11.12
F01D5.5
